# Into the wild: uncertain frontiers and sustainable human–nature interactions

**DOI:** 10.3389/fsoc.2024.1325963

**Published:** 2024-03-19

**Authors:** Jennifer Patterson

**Affiliations:** School of Education & Institute for Lifecourse Development, University of Greenwich, London, United Kingdom

**Keywords:** Earth ethics, deep ecology, paradigm change, eco-anomie, rewilding, sustainability, sustainable health

## Abstract

Humans seldom consider themselves as animals, and that humans are animals is a truth frequently turned into an insulting metaphor indicating “uncivilized” behavior in many cultures. Interestingly, the “civilizing” aspects of Western Culture in the Global North are historically derived from traditions of democracy based on living in cities from which the wild has been banished. This is embedded in the English language since civilizing and civilization come from the Latin for city, *civitas*, the place where citizens hold voting rights. Beyond the gates of civilization is the wild. How the wild and nature have been constructed and demarcated is an enormously complex and enduring challenge in western philosophy as it relates to knowledge-making, existence, truth, and reality. Indeed, whilst people generally believe they know what nature means, they rarely realize that little in nature is wild. Furthermore, the concept of uncertainty, central to the pandemic, is compounded by climate instability and a potentially disastrous future. This is breaking down what is known, requiring porous and flexible conceptual frontiers and a transdisciplinary approach. This article traces the linguistic separation of humans from their animal origins and wilder environments for political and increasingly greedy economic purposes. It explores the acknowledged complexity of healthy human–nature interactions, juxtaposing information mainly from the humanities and social sciences. Demonstrating how unhealthy the current paradigm has proven to be for humans and the natural world, it brings together conflicting information to disrupt traditional certainties using an innovative bricolage methodology. It weaves and combines different ways of knowing as it considers forms of knowledge-making, rewilding, foraging, the place of magical thinking, and vital force. It concludes that a new paradigm is needed to enable a way of working toward any vision of healthy human–nature interaction.

## Introduction

The French feminist philosopher, Irigaray (1999), has pointed out that we share breath with the Earth. Her lifetime of theoretical work engaging with the philosophical and psycho-critical implications of experience has moved focus from visual to inner, sensory experiences. She indicates the breath we share is an often-forgotten part of our lived experience, something that makes us one with nature (Irigaray, 1983). Yet, despite this lived experience, we pollute the air we exchange. The Earth's temperature is rising. We know this is exacerbated by gases caused by increasing human consumerism and that people living in more polluted areas are also becoming ill (European Environment Agency, 2023). Yet, while awareness of a pollution health crisis is increasing, air is rarely the subject of social research and theory and is almost always disassociated from the human bodies that breathe it (Allen, 2020). Such perspectives conflict with one another, and whilst being in nature, whether urban or rural, is enjoyed and considered good for people, not everyone considers a relationship to nature necessary for physical or spiritual health.

This conceptual article for the health and illness interactions special issue of *Frontiers* investigates the health of human–nature relations, using a bricolage methodology to review the situated knowledge of the Global North and consider how a language, research, and concepts of health have been constructed. It reviews counter-current theories and new directions before considering how to move forward. Crucially, global warming is a dire situation involving loss of the whole planet, a material reality that confronts different theories and positions on what is known, the linear past. Arguably, this requires new ways of thinking, recently described in the USA by Haraway (2016) as tentacular, forms of sympoiesis that move away from the Anthropocene (with people at the center of the universe). We are now in the Chthulucene, a “sort of time-place for learning to stay with the trouble of living and dying in response-ability on a damaged planet” with a networked, evolving universe of chattering critters or oddkin, creating stories on a warming dystopian compost heap (Haraway, 2016, p. 2). This article deploys standpoint theory (Hartsock, 1983; Haraway, 1988), poring over some frequently conflictual and often clumpy material in the heterogenic anthropocentric compost heap, where a plethora of historical and philosophical matter offers food for thought and indicates enduring beliefs. With its premise that all knowledge is socially situated and stems from a social position, this standpoint places emphasis on marginalized experiences, especially those of women. The premise of marginalization permits me to align my thinking as well as to consider the Earth as an exploited and marginalized entity in anthropocentric politics. Placing political and social power relations at the center, standpoint theory further supports my consideration of philosophical power relations embedded within disciplines (Haraway, 1988).

As a feminist critical thinker, I am particularly interested in the disruption of mainstream knowledge-making ideologies and the power they hold over other ways of thinking. I am influenced by the subversive and transgressive aspects of Harraway's work, where the compost heap forms a perfect analogy for the plethora of material. It is easy to get trapped in continually reviewing this dead material. It is incredibly hard to formulate a new position from which to speak, as Harraway does.

A linguist and critical theorist in French and Art History, I am an Irish white feminist academic working in education and an interdisciplinary researcher working across the humanities and social sciences. My interests include health, sustainability, and research methods. I draw upon the various methods that inform my academic background, including my professional practice as an integrated health practitioner and medical herbalist and my life experience as a person relating to the natural world. I make no claims to absolutes because the theories I engage with involve human and social worlds. Working with language and images across several disciplines with sustainability in mind, “thinking-through” (Ahmed et al., 2000) as much as “thinking-with-care” (De la Bellacasa, 2012) is part of the practice of transparency. For me, this is an activist political feminist engagement, a *practique* in Bourdieu's sense that it brings together theory and practice, behaviors and activism. Theorizing and conceptualizing what I see, feel, and believe are part of my passionate engagement with the natural world and my ongoing phenomenological engagement with a changing reality.

In my view, Western material worldviews are firmly embedded in economic and political ideologies of civilizing, rooted in Classical Greco-Roman culture, and fertilized by Enlightenment notions of truth. Language contains references to this anthropocentric politics of land ownership that positioned the natural world as a resource to be controlled, cultivated, and plundered. I believe this historic agenda, which includes patriarchal power dynamics and toxic masculine ideals, still influences contemporary capitalist human–nature relations. Furthermore, these environmental, political, socio-economic, and gendered implications persist and are embedded in academic research on the natural world. Indeed, as Haraway (1988, p. 205) states, “science as heroic quest and as erotic technique applied to the body of nature are utterly conventional figures.” Acknowledging that definitions of proof and truth entail conceptual complexity, I therefore examine how historically, mainstream culture placed the wild in opposition to it, a divisive practice unhealthy for maximal human flourishing that negatively influences human actions toward climate change.

For me, the recognition that the reality of living well in the Global North involves poisoning the Earth on which human life depends forms the starting point. To explore existing conflicts in this symbiotic relationship, I therefore question what “health” and “living well” mean. This requires reflection on how migration from the country into cities at key political and economic points historically underpins Western civilization and governance. I suggest that where a land-based belonging forms part of many peoples' construction of identity, place forms an anchor for existence, and being-in-the-world is an existential crisis that sits behind discourses of “dis-placement” faced by many people forced to migrate. Yet, outsider identities demonstrate that whilst citizenship identity is mainly state- or place-based, these are entangled political and social topics (Yuval-Davis, 2004; Nordberg, 2006). I have also been influenced by a recent video for the Association of Medical and Healthcare Humanities (AMHH), in which Columbia's first indigenous Ambassador to the United Nations, Leonor Zalabata, discussed how place is central to identity and conveys healing for the Arhuaco people (Patterson, 2022). I had not considered place as having a healing role other than in relation to plant species and phytochemical constituents, and yet for me, this is a critical part of my Irish identity. I am indebted to Zalabata for this insight. In this article and thinking of place and displacement, I particularly consider how mainstream culture has not only separated people from their animal identity but also from a sustainable, ethical care for the Earth. This implies a relationship with nature is necessary for improving physical, mental, and spiritual wellbeing and for lived experience, a phenomenology that gives life particular meaning. Yet, whilst I consider existential, spiritual, and physical identity, I also recognize that climate change is a material reality and not something that can be controlled by humans. Physical events bring death, injury, starvation, and trauma, and climate anxiety is an increasing mental health issue (Hickman et al., 2021). Faced with unknown science, it is my contention that all knowledge becomes theoretical and requires imagination to “perceive” any future.

To get to this space, I review and deconstruct existing culture and knowledge in the form of language, research and mainstream, philosophical counter-currents, and contemporary actions, uncovering a diagnosis of eco-anomie. Bearing in mind again that the matter in the compost heap is text (from the Latin *texere*, to weave), what I do here is to unravel materials, texture, and threads. I contend that the process of dissecting and reframing presents a systematic and rigorous rationale that identifies the pathological damage of the Anthropocene as societal eco-anomie, a profound un-knowing that results in stagnation or stasis. I aim to demonstrate how the current paradigm devalues the relational systems necessary for human health and healthy human–nature interactions. Metaphorically raking over her compost pile to generate heat, Haraway (2016) argues that stories in the Anthropocene end badly, and she generates new ones, refiguring new myths for possible futures that storytelling brings into being. For me, reconnecting with the earth is essential for human wellbeing, for working with uncertainty, and for envisaging any possible future, with or without humans. In this article, I use bricolage as a method that repurposes and changes the materials.

Proposing that it is necessary to step “into the wild” I am calling for a paradigm shift (Kuhn, 1962). By “staying with the trouble” alongside Haraway (2016) and reviewing counter-current human–nature ideologies, my arguments clarify the need for this metacontextual shift. Thus, my call to improve healthy interactions from within the humanities and social sciences echoes calls for reframing the field of ecological theory to support eco-criticism (Glotfelty and Fromm, 1996) and those from environmental observational research by scientists such as Will Steffen (Boonstra et al., 2023). Through sustained arguments, I conclude that an Earth-centered paradigm with inputs from many disciplines (Rigolot, 2020) and transgressive practices, premising the Arts is needed to bring healthy human interactions together with those of an unpredictable, dynamic, and changing planet. Imagining creates hope. Located at the crossing places or boundaries of disciplinary mainstreams, I occupy a hedgerow-space where wild things flourish outside of cultivated and well-plowed fields. From this space, I sketch out the new paradigm.

I acknowledge their fractious perspectives, methodological challenges, and limitations of the disciplines I draw on, as well as the impossible size and complexity of the topic. The selection and use of materials necessarily present my partial views, a perspective supported by using standpoint to clarify the politics of place-based situated knowledge in the Global North (Haraway, 1988). Used flexibly here, this standpoint further supports my researcher's insider expertise and outsider engagement with various disciplines, and my readings and juxtapositions of common philosophical episteme together with intersectional differences in lived experience (Wylie, 2003).

## Methodology and concepts

In this article, I build a theory located within a transformative social constructivist paradigm. Drawing on different disciplines, it includes real-life practicalities aiming to produce something that is innovative and fundamentally transdisciplinary (Rigolot, 2020). It is a makery that involves “bricolage,” a DIY methodology of taking things apart, examining them, and reassembling them. It deploys this creative and philosophical method for diagnosing cultural production. In social sciences, bricolage was conceptualized in 1999 and elaborated by Denzin and Lincoln (2011) as an epistemological research methodology, extensively explored in 2001 in the work of Kincheloe et al. (2011), and discussed in Rogers (2012). It is associated with qualitative methods that combine to represent whatever is being researched from different perspectives. Bricolage supports methodological transgression, is useful for pushing boundaries and for a politics of including unusual material. It suits the selection and deconstruction of materials used here. The process of recycling, upcycling, and repurposing for future use correlates with the aims of the research. Important for reframing its methodological use, bricolage has different origins, not only in social anthropology but also in art.

For the French anthropologist Lévi-Strauss (1966), bricolage is not imaginative since what is built is pre-conceived. He named skilled craft work using bits of leftovers, “bricolage,” differentiating intuitive knowledge and wild or uncivilized knowledge in place-based and plant knowledge as magical thinking (Lévi-Strauss, 1966). This signifies making do with what is to hand; the French verb *bricoler* means fiddling about with leftover materials. Bricolage is an analogy for mythical thought (Johnson, 2012). Levi-Strauss contrasts the bricoleur (maker of myths) with the engineer (modern science). In complex arguments, Derrida (1967a) counters that the bricoleur has created the engineer since the materials (signs and structures) pre-exist and become decentered because meaning and knowledge-making shift.

The innovative and transgressive bricolage methodology I use here explicitly incorporates both social science and humanities aspects. Bricolage references the found art of Dada and Surrealism, whose conceptual dislocations have origins in Modern Art as a visual metaphor for viewing dislocated or distorted planes of color dating back to the nineteenth-Century French Impressionist painter Paul Cézanne (1839–1906). When he becomes interested in or moves around something, it is enlarged out of proportion to the rest, as, for example, the statue's foot in his painting *Still Life with Plaster Cupid*, c.1894 (Courtauld Gallery London). Cubism's distortions exaggerated this process and were followed by dislocations of time and space in de Chirico's art. These processes influenced the Surrealists, the theories of Lefebvre and the Situationalists and later urban films (Patterson, 2014). Surrealist poems also use grammatical structures to bind together words and images (signs) that do not logically belong together. The anti-art premises of Dada's found objects led to the surrealist incongruity of placing things together that did not usually appear together (famously a sewing machine and an umbrella), creating a different sense of reality and challenging what “art” means in terms of content. From the 1960s onwards, drawing on these origins, the *arte povera* movement actively reinforced the creative sense of the bricolage technique. More recently, Fortais (n.d.) described the bricolage technique used in her Ph.D. (2013–2018) at the Slade School of Art as “disassembling and repurposing.” In art, the creative aspect of bricolage generates something new and innovative. Making new ways of putting things together possible, bricolage can present an original and creative way of talking about something, generating a postmodern form transcending Levi-Strauss' structuralist anthropology (Hester, 2005). Furthermore, a contemporary reading includes the current everyday sense of DIY doing home repairs associated with commercial outlets such as “Monsieur Bricolage.”

I embrace this transgressive aspect of bricolage that is allied to innovation. The methodology incorporates its multidisciplinary ontological premise to emphasize the need for disrupting unilateral perspectives by a purposive selection of materials from a wide range of sources on human–nature relations. Examining and deconstructing these materials, it aims to disrupt by moving beyond traditional perspectives, changing and upcycling information aligned to a different, more sustainable purpose as it considers how to place the new material. The process focuses on bits and pieces of information that serve intention and arguments rather than aiming to be comprehensive. As I aim for fluidity in a creation that may necessarily sketch ideas and thoughts, I acknowledge its limitations as problematic barriers I step over, for now.

Deconstruction, and particularly Foucault, form a secondary methodological consideration. Foucault (1969) engaged in radical social critique by tracing power and control in Western Culture and institutions. Philosophically, (Foucault, 1988) late work moves beyond poststructuralist ontological containment associated with Derrida's (1967b) deconstruction techniques, by pointing to two possible types of truth: the truth of life and that of lived life, or life experience. These final lectures at the College de France examine cultural technologies for developing knowledge, classified as production, sign systems, power, and the self, the latter critical for transformation. Foucault's few references to nature emphasize its role in the spiritual dimension of human identity. He considers how self-examination (reflection on experience) historically supported ways of dealing with future misfortune and uncertainty, arguing that since the Enlightenment, material and spiritual culture sit in opposition to one another, placing people in a dilemma and confusing what it means to be human, to know oneself, and to live well.

Although the bricolage used in this article did not intentionally follow a Foucauldian pattern, I certainly sampled mainstream language (signs), research (knowledge-making process), and counter-current theories in consideration of knowledge. I use reflection and occasionally discourse, and there are other parallels. Living well, elements of standpoint, positioning, and Haraway's work align with my main arguments. Returning to Foucault, care of the self requires revaluation of a human spiritual dimension that includes the natural world, a conclusion echoed here. Aiming for transdisciplinary transformation, I am adapting critical and social theory tools, including aspects of feminist theory, to inform my deconstruction and reasoning as I walk firmly into the agentic wild. Here, a sensory aspect forms part of experiential and relational connections between people and nature. This permits reference to other ways of knowing, intuitive practices, and shamanic aspects of different cultures and traditions. What follows then is an analysis of material that I have gathered with respect to human–nature interactions. It references first “mainstream culture”, and second “counter-currents and new directions.” In my discussion of mainstream culture, I consider ideas embedded in language, looking at words such as “nature,” “wild,” “soil,” and “Earth.” Consideration of mainstream knowledge production through research in relation to health and nature interactions follows. A proposition about the health of mainstream human–nature relations concludes the first section. In the second section, counter-currents and new directions, I consider ideas about vitalism, biophilia, deep ecology, and rewilding. This is followed by a second set of propositions. My call for a paradigm change brings together the main strands of the work.

## Mainstream culture

### Language, diagnosis, and human–nature relationships

Human–nature interaction is worthy of critical social investigation since at its heart sits a discussion on cultural reproduction. Civilizing practices created a damaging dualism, including the extensive “nature” vs. “nurture” debates whose origins go back to Classical writers with their underpinning ontology of “truth.” Mythological and lived examples include feral or wild children: Romulus and Remus in Ancient Rome; Truffaut - Truffaut and Berbert's (1970) film *L'Enfant Sauvage*; and the abusive 1960s case of Genie (b. 1957) in the USA. This binary between civilized and wild shifted humans from mammal identity.

### Language: nature

Trying to define “nature” demonstrates that something seemingly familiar is incredibly complicated. The first session on my outdoor learning course involved students being in “nature,” in the park by the campus (without phones), observing their surroundings, clouds, and trees. Only one student ever commented that this was “managed nature” or discussed what they believed “nature” to be. Taking a step back, it is important to question from the outset what “nature” is and to clarify its socio-linguistic construction.

Consider what this often-used word means in a concrete sense. Whilst most would agree that nature is part of being human and humans are part of nature, being “an animal” has a long derogatory linguistic tradition. Calling someone “an animal” is an insult. Historically, this animal aspect has been negatively associated with showing emotions, with women, with female sexuality, and with a shameful animalistic feminine (Nussbaum, 2006). This may partly account for a substantive feminist disengagement with nature (Alaimo and Hekman, 2008). Moreover, as early Earth worship in female form (Gimbutas, 2001) and subsequent patriarchal subjugation (Lerner, 1987) presented a binary sexual politics in second-wave feminist culture, navigation is complex. Yet, as Alaimo and Hekman (2008) comment, the separation of people from nature has not only harmed women but also indigenous peoples and other marked groups.

Ducarme and Couvet (2020) demonstrate how the concept of “nature” appears as a later translation of Classical Western texts. Etymologically formed from the root of the Indo-Germanic “be” or “being” in the sense of “grow” and “born,” nature is an ambiguous concept. Aristotle struggles to define whether it is form or matter, substance or vital property, concrete or abstract principle. In Roman translations of his writings, “nature” involves the complex essence of things, as, for example, in the phrase, “the nature of plants and of humans” or it was “her nature.” So, in its early sense, the nature of a thing is its essence, whether it is plant, human, animal, or natural environment. Later, Cicero introduces the nature/culture opposition. This complex, enduring philosophical debate is critical for thinking about humans and existence. In the global North, “nature” carries these patriarchal western cultural origins.

Ducarme and Couvet (2020) describe how “nature” has been further subjected to religious appropriation and material realism. The nature/culture opposition frames the “nature” vs. “nurture” debate running through educational theory and extending today into genetic science. Art and literature romanticized the natural world during the eighteenth and nineteenth Centuries. Rousseau's political work *Du contrat sociale* (1762) underpins modern rights discourses and the constitutions of republics such as France, the USA, and Australia. Meanwhile, his work *L'Émile ou de l'Éducation* (also 1762) locates the ideal education for a (male) moral citizen as being brought up in nature to avoid social corruption. These wide trends of real and ideal representations of nature informed the work of artists, writers, and even gardeners such as Capability Brown (1716–1783). Simultaneously, the enormous classification projects of the eighteenth-Century Enlightenment presented new systematized ways of categorizing (and dominating) the natural world, eventually placing the word “science” in opposition to nature and observational study. Formerly a core scientific concept, nature is clearly a concept changing over time that is entirely socio-culturally created and embodies the knowledge-making ontologies that it intersects.

Moreover, as knowledge (*scio*) became more closely associated with education, other ways of knowing, such as intuitive, kinaesthetic, and sensory understandings, became devalued in mainstream culture. The social anthropologist Lévi-Strauss (1966) privileges science over indigenous knowledge of nature, which he calls “*sauvage*” or “wild,” rationalizing it as magical thinking. I grasp this term with gratitude. There is something linguistically magical about an intangible, familiar, and unaccountable idea of nature that comforts me. Its existential aspect has been abstracted and refracted. Its meaning is discontinuous and fractured (Larrière and Larrière, 2015).

In mainstream culture, nature has become a phenomenon that is known to exist but hard to define. Yet it is an attribute that humans share with the wild. Even as a cultural creation, nature is still essentially understood to be alive and to involve a living force. Today, it is neither a scientific term nor a philosophical concept. Its conflicting meanings only sometimes include people. Importantly, whilst widely understood, the word “nature” is used in entirely different ways in different academic disciplines, from philosophy and literature to ecology, conservation initiatives, and climate change policies. So, there is a political necessity behind the call for linguistic socio-critical consideration of words such as “nature,” which includes recognizing variability if they are to be used from an eco-centric perspective.

### Language: polite, civilizing, and urbane…

Stepping further back, the words for politics (*polis* in Greek), civilization, civilized, civilian (*civitas* in Latin), and urban, urbane (*urbs* in Latin) refer to the origins of democracy in Classical city-states. These were individually organized defensive structures and units of government, with aristocratic hierarchies and slavery. Rural nature was farmed to provide for citizens. Indeed, the word “economy” from the Greek (*oikos* meaning household and *nemein* meaning management) originally referred to natural abundance. It incorporated ethics and the frugal management of a wider estate, in the sense of managing agriculture (land) and slavery (people) for ethical purposes or a good life (Leshem, 2016). During the first 400 years of the Roman Republic, lines were drawn at the gates of the city, and only citizens could vote. Domination over the wilderness demonstrated power, particularly imperial power. Citizens were protected from the wild, which was presented as spectacularly fearful and unpredictable. Wild animals were brought to Rome and killed in the Colosseum for propaganda and entertainment. Elsewhere, I have written about contemporary regulatory practices that grew out of identifying “risk” in Medieval times. This new word describes nature's unpredictability and a fear of it interfering with commerce and the delivery of goods (Patterson, 2017). Material culture is built on changing ideas of the economy for growth or profit. The insurance business was created to avoid unpredictability. The English colloquial phrase “beyond the pale” means being unpredictable beyond civilization, in reference to the area beyond the ditches surrounding the city limits of Dublin, Ireland. It is still used to signify impolite behavior. Underlying these examples is a message of wilderness subjugated in both animal and human form.

### Language: wild

Associated with words such as *weald, wold*, and *welt*, the word “wild” comes from Saxon, Norse, and proto-Germanic origins, signifying early historical invasions marked in language. Much like the word “nature,” “wild” has been negatively associated with something that is not under control, with uncontrollable sexuality and emotions, primarily with women but also young men. The etymology of “weed” from Anglo-Saxon is allied with words such as *weald*, which may reflect similar meanings of wilderness or plants taking over the landscape. Today, many plants considered weeds, such as dandelions or nettles, are valuable herbal medicines, whilst phytochemical constituents in others have been used to create an estimated over 50% of mainstream medicines (Gurib-Fakim, 2006). In English, there is a broad division between words from Old French, encompassing those associated with courtly (cultivated) behaviors from the incoming French-speaking William the Conqueror in 1066 and over 500 years of ruling nobility. Hence words such as baron, manor, royal, court, dance, chivalry, and the words for meat such as mutton, pork, and beef come from French. Meanwhile, the farmer, being local, spoke Old English (Anglo-Saxon and Germanic origins), using the names of the animals, for example, sheep, pig, and cow. It is not surprising that etymologically, “wild” sits outside of civilizing premises. Another Old English word useful for the arguments in this article is “heal,” which literally means whole.

### Language: soil, dirt, and Earth

In the USA, the material that covers the planet is called “dirt.” I have observed children at Forest School terrified of becoming “dirty.” UK English uses the word “soil” rather than Earth. Whilst they are synonyms, soil also means dirty, where Earth and Earthy have life-supporting, healthy associations. Earth is now more commonly used to describe the planet, yet Earth-writing is writing of the soil, comprising a politics of laboring and a visceral sensory engagement linking past and future (Griffin, 2023). Human manure was the Victorian “night soil” collected and taken to fields where it was spread as fertilizer. This is still the case. Today, Britain is known for the adept re-use of sludge cake (Sustainable Food Trust, 2015). Yet a large study (Wilkinson et al., 2022) across the globe found high levels of active pharmaceutical ingredients, specifically carbamazepine, metformin, and caffeine, polluting rivers across the globe, highest in lower to middle-income countries, and posing threats to human health. Carbamazepine is associated with epilepsy and diabetes, and metformin is a well-known antidiabetic pharmaceutical that disrupts the endocrine system. UK rivers showed higher pollution levels than other European rivers. People ignore how the increasing use of processed food and fertilizers is affecting agrobiodiversity (Leite et al., 2022). Dietary issues are linked with current health issues, especially for those on lower incomes globally (World Health Organisation, 2023). Even without pharmaceutical pollution, food can make us ill (excessive sugar), provide nutrition for health, or act as medicine, one example being beetroot for blood pressure (Bonilla Ocampo et al., 2018). Meanwhile, soil feels tangible, and the Greek root of the word gives the chthonic origins for Haraway's ecological shift to the Chthulucene compost heap.

### Research: benefits and behaviors in human–nature interactions

Research in health is driven by illness and treatment or medicines. Only recently has research around human–nature connections begun to evidence wellbeing and health benefits. Chawla (2020) reviews methods and measures deployed for children and Beery et al. (2023) for adults. Meanwhile research evidences a positive correlation between adults' active care for the environment and significant time spend in nature during their childhood, something needed for the future of caring for the earth (Chawla, 2007).

### Research: children

In the UK, Margaret and Rachel McMillan (Moriarty, 1998) created outdoor nursery camps over 100 years ago to address children's illnesses from pollution and poverty, harms that persist today. Currently, outdoor freedom includes stranger danger and a cultivated fear of nature in literature. Louv (2005) pathologized “nature deficit disorder” with symptoms of rising anxiety and decreasing knowledge and understanding, like thinking food comes from supermarkets and eggs from cows. The United Nations Convention on the Rights of the Child (United Nations, 1989) gives children a right to natural behavior (play) but no right of access to nature. Yet, in the Global South, time can be an unaffordable luxury, and nature is physically dangerous. Many children must spend time growing food in fields rather than going to school. However, four indigenous Huitoto children survived a plane crash in the jungle for a month thanks to their knowledge of fruits (Youkee et al., 2023). In the Global North, it is increasingly clear that children experience benefits from time in nature, developing risk-mediated behaviors, fine motor and social skills, as well as knowledge (Murray and O'Brien, 2006, 2007; Chawla, 2020). Dillon et al. (2006), like Louv, found significant barriers. Children are afraid of outdoor settings. Such findings underpin changes in early childhood education in the UK (Forest School Association, 2023). Meanwhile, multicultural identities need consideration; the existential challenges of UK nature for different ethnicities are rarely considered.

### Research: adults

In Japan, research found that *Shinrin-yoku*, or forest bathing, has beneficial physiological and psychological effects, especially for people with depressive tendencies (Furuyashiki et al., 2019). Shanahan et al. (2016) used a nature-dose framework (intensity, frequency, and duration), finding that long visits to greenspaces correlate with lower rates of depression and high blood pressure and more frequent visits with better social cohesion. In a randomized control trial (RCT) with 30 allotment gardeners, Van Den Berg and Custers (2011) demonstrated that gardening reduced biomarkers of stress (cortisol) more strongly than reading. Research like this now informs social prescribing, such as gardening, by qualified UK GPs for mild depression and other illnesses.

Ulrich's (1984) frequently cited research compared a window view of nature vs. one of a brick wall for gall bladder surgery patients. Obtaining 23 matched pairs of patients from 9 years of records, we found statistical differences in recovery rates, pain medication, and longer-lasting positive mood changes vs. negative feelings. The research is interesting yet problematic; the data are retrospective, with different nursing staff reports on mood, unconscious allocations, and other variables.

The UK Office for National Statistics (Office National Statistics, 2021) reported the impact of lockdown on peoples' relationship to nature, visiting parks or playgrounds, outdoor attractions, or beauty spots. However, the focus was on exercise rather than the type of environment. Natural England's (2021) “People and Nature Survey” (PANS) aimed to establish the impact of COVID-19 on people's engagement with green spaces. Building on 10 years of data, it used a panel method for continuous sampling of up to 25,000 adults. The study combines a survey with segmentation of questions and social media analyses. PANS found that visits remained consistent. Confirming earlier studies, affluence (household annual income > £50,000) was a factor. Disadvantaged households visited green spaces less frequently. People from minority ethnic groups and those on low incomes were less likely to access nature, especially blue spaces, and young peoples' wellbeing was more likely to be impacted by lockdown. Higher wellbeing and happiness scores indicated a greater likelihood of visiting green spaces for physical health and exercise than those with lower scores, for mental health. Interestingly, different ethnicities visit different spaces, and particular environments demonstrate gender affiliations. The detail in the study demonstrates that human–nature interaction is socially and culturally construed.

Vespestad and Lindberg (2011) consider nature experiences within the tourist industry using an ontological framework. They identified four categories where nature is central: a setting, a state of being, or a means of framing identity. Their marketing slant focuses on consumer desires. It highlights something I call “nature-tainment” from “nature” and “entertainment” that requires unpacking elsewhere but ranges from managed nature “bubbles” that exclude animals but where people can stay, through conservation-type experiences, to attractions that are far from natural and where nature simply provides a backdrop. Finally, a growing body of research, considering whether stimulated nature can positively affect human health, demonstrates positive results (Depledge et al., 2011; Browning et al., 2020).

### Digging deeper: health, health culture, and healthcare

Concepts of health, wellbeing, and illness are framed and challenged by what health means in biomedical terms. Engel proposed his inclusive systems-based biopsychosocial model over 40 years ago, yet un-ease between different world views about the body of a person continues in tangible and intangible entanglements (Patterson and Kinchington, 2019). The current and evolving bio-psycho-social UK medical model emphasizes the interplay of different factors (Alonso, 2004; Gifford, 2016; Gask, 2018). However, the transition from the traditional science-based medical model (driven by STEM, science, technology, engineering, and medicine disciplines) to the biopsychosocial model (influenced by the social sciences, humanities, and the arts for people and the economy, or SHAPE) has been slow and uncomfortable (Imafidon and Black, 2023).

Medicine, or biomedicine, is described by Sontag (1978) as a country with its own language, culture, and inhabitants. Financially, it is the dominant form of healthcare globally, rooted in western knowledge-making and market-driven practices and dominating indigenous and other medicines. Historically, numerous treatments have been wrong, including thalidomide drugs and the various re-categorizations of trauma, previously seen as genetic weakness. Meanwhile, specialization into different body systems has created distinctly siloed fields. In humanities and social sciences research, reading data out of context is a serious ethical issue, yet this is how biomedical research into human bodies and biomedicines has functioned until recently.

The National Health Service (NHS) works with illness, not health. Patient waiting lists are long. High staff attrition rates (Finlayson et al., 2002; Lock and Carrieri, 2022) portray an institution at breaking point. Burnout is common, race discrimination continues (NHS, 2023), and leadership is problematic (Messenger, 2022). Iatrogenic causes (doctor-related) are the third leading cause of death in the USA (Greger, 2015). For O'Mahoney (2016), kindness has become sclerotised. If illness is the “night side of life” (Sontag, 1978, p. 3), death is even more problematic. Medicine has become “a service industry” with death, something people and society hide from (O'Mahoney, 2016, p. 270). Yet death is as important as life; it is part of being an animal and of spirituality.

Advancing technology has brought earlier diagnoses, and it confirms how illness is connected to the patient's body as much as their experience. The application of systems theory from land ecologies signifies an exciting shift in viewing bodies as interconnected living systems allied to the natural world. The gut, tongue, colon, and intestines are now called microbiomes, each with individual communities of bacteria. In previous centuries, medicine was underpinned by a holistic view of the body, treating the whole person to achieve “healing,” meaning “whole.” Mind–body connections in specializations like psychneuroimmunobiology demonstrate how psychological and behavioral modalities affect physiological health (Irwin, 2008). Recognition includes personalized medicine, environmental medicine, and social prescribing. Crucially, happiness and wellbeing influence physical health.

The best prevention of illness is health and wellbeing, yet “health” research funding is geared toward restoring, not sustaining, health. This goes some way toward explaining the paucity and methodological challenges in researching healthy human–nature relations. Currently, living “a good life” in the Global North involves over-consumption that contributes to illness throughout the globe. Moreover, the disconnect between notions of health and illness arguably affects personal agency and healthy practices. Rethinking medicine as a whole system for sustainable health requires a transdisciplinary dynamic. It's important to consider whether biomedicine has moved too far away from the human animal and from the plants and foods from which much of our health originates.

### The health of mainstream human–nature relations: eco-anomie

Most eco-critical human health research comes from a perspective where nature is benign and therapeutic. Large predators are long extinct, and danger is predictable. Yet, every year, people die from being in nature. For some, this is a lack of real-world knowledge; others underestimate individual fitness levels or enjoy dangerous challenges (Gatterer et al., 2019). It is as much about not-knowing as existential values, since there is evidence linking addiction to extreme environmental challenges and psychiatric disorders (Habelt et al., 2023). Micro-level stressors in the immediate environment negatively influence mental health, and evidence is growing for macro-level stressors, such as climate anxiety. Health, illness, and wellbeing in human–nature relations are worthy of further exploration.

There is less recognition in the temperate Global North of different perceptions in the Global South, which experiences extreme climates and life-threatening conditions such as poverty, war, famine, and flooding. Glaring economic and societal imbalances demand reflective critical race theory. Meanwhile, politicians enact an unhealthy mix of blame and shame around pollution, child labor, and consumer culture. Literature on topics such as climate change, fashion, and conservation by writers and journalists such as Naomi Klein, Lucy Siegle, and Jo Mooalem, evidence and question the global environmental and social consequences of real-life consumer production processes and behaviors. If environmental problems are a known by-product of the Western civilizing culture of the Global North (Glotfelty and Fromm, 1996), the existential human–nature identity dilemma that separates people's existence from ethical and spiritual understandings, polarizes arguments. Seeing people as human animals places them closer to nature and uncertainty within an unstable, terrifying (alien) environment.

Drawing on social theory and particularly on Durkheim (1858–1917), I use the term “eco-anomie” to describe the separation leading to this breakdown in the human ethics of Earth management (the Ancient Greek *eco-nomie* discussed earlier). For Durkheim, “anomie” (relating to labor relations and disconnection between those who work with the soil and professional bureaucratic classes) exists in balance with understanding of cultural norms or “nomie” (Marks, 1974). Today, capitalist material consumer culture is upheld by wealthy people who demonstrate cultural and societal nomie and mostly profound eco-anomie.

The French social theorist Pierre Bourdieu (1930–2002) talks about habitus, capital, the field, and practice or *practique* (behaviors). Capital is usually added through education and carries cultural value, whilst habitus is more about personal and informal traits. Human behavior (*practique*) comprising a powerful consumer politics of greed has propelled human–nature relations out of balance. Contributions to climate change initiated in the Global North also constitute a profound political eco-anomie (un-knowing or ignoring) because what poisons the Earth also poisons the animals that live on it, including people. Indeed, this *practique* increasingly suggests other forms of disconnection negatively affecting health: poor diet, processed foods, lifestyle, and stress. Eco-anomie is a loss of habitus that can only be partly addressed through education.

Eco-anomie has caused an increasing mental and physical dis-ease, a new sort of autoimmunity, and the alienation of self. A 2023 study shows that clinically diagnosed autoimmune disorders now affect 1 in 10 people in the UK. The authors have concluded that some people are more susceptible (or less immune) to environmental and social risk factors (Conrad et al., 2023). Modern diet and lifestyle are important contributors (Greger, 2015). Indeed, the structures involving intracellular immune receptors that recognize pathogens, causing immune system cascades and ultimately cell death, are similar in plants and mammals (Mermigka et al., 2020). The autoimmune split between people and nature is a disease resulting in disorder, withdrawal, and stasis in actions to address climate change. Fear and anger generate extreme positions. In this, I understand alienation as a form of “othering” and autoimmunity as a metaphor comparable with Derrida's poststructuralist concept based on rational identity (Rae, 2022).

Eco-anomie, the un-knowing of healthy human–nature relations, is a result of culture-generated divisions between humans, nature, illness, and health. Loss of connection to nature frames disruption of identity and dislocation of reality. Eco-anomie further creates loss and separation in human intersubjective space and body and place-based disconnection in the sense of Lefebvre's Situationalist urban journeys and dystopias (Patterson, 2014). Healing, or the wholeness of human–nature relations, is required for a healthy balance. In this sketch, I acknowledge the need for further elaboration on the social and environmental complexity of eco-anomie.

### The state of relations

Diagnosis of eco-anomie in mainstream culture means people are relationally disconnected, and lack agency and knowledge for connecting themselves to nature, the Earth, and uncertainty. Societal “norms” have wiped out agency. This is a disease in life, an autoimmunity, with increasing mental and physical effects. Current anthropocentric frameworks and terminology for knowledge-making are academically and culturally protective. Eco-anomie is profoundly harmful to human flourishing, yet it is a disease inimical with any form of biomedical “treatment.” A new paradigm is necessary to begin to improve homeostasis and restore balance. Innovation in the field of ecological theory within an Earth-centered paradigm may support a degree of healing but cannot undo the damage. The next section searches for hope as it considers alternative theories for human–nature relations.

## Counter-currents and new directions

An intermittent parallel discourse of positive human–nature relations is entwined with mainstream culture (Glotfelty and Fromm, 1996). Important historical ontological premises around spiritual and mental health include pagan nature spirits and the importance of gardens (and wilderness) in all major religions, as well as philosophical inquiry around what it means to be human. Environmental markers include vitalism, deep ecology, and the psychological concept of biophilia, briefly outlined below and followed by a critique of rewilding, a currently popular practical solution.

### Counter-currents: vitalism, deep ecology, and biophilia

Originating in Ancient Greek philosophy, vitalism is a metaphysical paradigm that holds that there is a hidden vital spirit, energy force, or soul in every living thing. Vitalism was accepted thinking in observational science until Cartesian dualism placed it in opposition to material science in the seventeeth century. Its main concepts, including homeostasis, or a healthy system being in balance, underpin integrated medicine and Gaia theory today. Coulter et al. (2019) discuss vitalism's history and philosophical debates in healthcare, calling for a rethinking of its application. As a principle of life processes, it may offer ways of thinking about treating imbalance in patients as much as human–nature relations.

Deep ecology is a term coined by Arne Næss in 1973 that has been called a revolutionary ecology movement (Devall, 1980). Predicated on twentieth-century authors including Rachel Cason and James Lovelock, deep ecology bears a clear relationship to the approach in this article. It incorporates environmental ethics, refutes an anthropocentric approach, and involves self-examination of behaviors and beliefs. Critical of nature being viewed as a collection of resources, deep ecology is a gestalt person-in-nature philosophy, viewing people and nature as one and looking to reform values and social organization (Devall, 1980). Today, an increasing amount of literature demonstrates its counter-current vitality. The literature is sensuous, poetic, and reflective, evidencing rich animal-in-nature experiences. In Monbiot's (2013, p. 230) *Feral*, taste sensations of cockchafer larva breaking on his tongue recall memories, and he “porpoises” whilst diving for dinner during spider crab migrations, in a world that he “could give [himself] up to.” Abram (2010, p. 230) feels the “enfleshed body” as a “sensitive threshold through which the world experiences itself.” Tree's (2018) intimate experience of rewilded land belongs here too. Deep ecology identifies the creative joy of human beings as nature, evidencing possibilities and hope.

Proposed by Fromm in 1964 as a human personality trait or psychological orientation toward life, biophilia was further developed by Wilson in 1984, meaning people form a mental link with living organisms. Wilson stated that this was something evolutionary, involving emotional dependence. Reprising the origins of biophilia, Barbiero and Berto (2021) argue it can refer both to a love of life between living creatures and to a love of nature, including animals and the environment. Perhaps because it reads almost as a diagnosis, the authors evidence how Fromm and Wilson understood this as a basic human force for developing balanced relations between people and the biosphere. They suggest it may be a fundamental evolutionary adaptation, with wilderness remaining deeply imprinted on the human psyche. Indeed, as Mooalem (2014) points out motivation drives some people to rescue animals in danger of extinction to extraordinary lengths, dressing as giant birds to support migratory patterns, for example. Barbiero and Berto cite an excellent range of research on positive emotional and psychological benefits such as happiness, relaxation, and wellbeing for adults and children in support of human biophilic nature relations as a rationale for biophilic design in human environments. The marketisation of the emotional value of human–nature interactions, including corporate use of “greenwash” (Gatti et al., 2019), would benefit from the further critical multidisciplinary study. Biophilia is in part a psychological spin on deep ecology and evidences the academic need to rationalize (and separate) some peoples' need to connect with nature.

### New directions: rewilding

More recently, Rewilding has emerged as a practical response to rural estates generating a new form of economy that recognizes that “nature” should be in charge (Tree, 2018). Initiated in Europe, Global Rewilding (2023) has formed partnerships focused on animal and land conservation. Rewilding functions differently in agriculture and conservation. Conservation usually involves the preservation of “wild spaces,” something that is frequently accompanied in the UK and the USA, more than in Europe, by a fear of “letting go” (Monbiot, 2013, p. 226).

Writing about the rewilding project at Knepp, in Sussex, Tree (2018, p. 61) concluded that scrubby pasture populated by large grazing herbivores is nearer the mark than previous understandings of “closed-canopy woodland,” correcting longstanding conservation mythologies of a tree-covered Earth before human intervention. Tree (2018, p. 153–154) argues that the term originates in the USA (citing Forman for its first use in the 1980s), comprises “a Pandora's box” full of idealist traps, and warns about “playing loose” with it. Academic descriptions of rewilding reference “cores, corridors and carnivores” (Soulé and Noss, 1998) and “a type of large-scale biological and ecological restoration, emphasizing the recovery of native wide-ranging species, top carnivores and other keystone animals in natural patterns of abundance to regain functional and resilient ecosystems” (Johns, 2019). Examining the literature, Carver et al. (2021) found 10 rewilding principles, including native species, the need for iterative feedback, and a transformative form of co-existence that he suggests establishes a shift in established relations.

Rewilding is a large-scale cultivated response, driven by landowners or those working with land (Rewilding Britain, 2023a). Informed by discourses of hope in mitigation of the climate crisis and by crises in agriculture, it is an emotive topic (Tree, 2018). Consumers and supermarkets in the Global North demanding cheaper production, perfect produce, and year-round availability drive agricultural production, especially in poorer countries with warmer growing conditions. Production requiring water and fertilizers results in over-production of food waste, animal waste (slurry), an increased carbon footprint from transport, poor working conditions, desertification of land, water shortages, and pollution of rivers, reservoirs, and oceans from fertilizers and plastics. Responses include local sourcing, box schemes, and organic and regenerative agriculture, but there are cost implications.

Where rewilding takes agricultural land out of production, it both reduces the carbon footprint and stores carbon. A huge expansion is needed for a substantive impact on carbon emissions (Monbiot, 2019). Rewilding supports greater and much-needed biodiversity. Technically, the essence of nature is a wildness that transgresses managed spaces. Recently, on a well-known gardening programme, the panel was asked if they would pay to see weeds. Disparaging responses included words like “untidy,” “anarchic,” and “native weeds.” Nobody pointed out the irony of getting in a petrol-driven car to visit an attraction that aims to reduce carbon emissions. Crucially, in their animal form, despite large predators, people are noticeably absent from rewilded landscapes, whether they are apex predators or prey. At Knepp, people walk on paths, occasionally crossing public rights of way. They are found in the café or the shop. Wider behavioral change is needed in the face of climate change. It neglects social considerations, places where people work with land for their survival, and the politics of global social diversity and critical race theory.

The question is whether rewilding can be more than an anthropocentric, affluent, and mainly Eurocentric fashion, encouraging nature to take over un-managed spaces like my hedgerow. Rewilding is clearly a force for good. It is an enormous educational opportunity to reconnect people with nature's “vital force” and learn about flourishing and unpredictability. It communicates life principles, offers space for actions, and evidences people's need to act. It accesses people's love of nature, their emotional need to reconnect, and drives agentic, small-scale individual and community actions. It has both reach and popularity. It is fast becoming a business, spawning books, educational work, and marketing that proposes to rewild everything from window boxes to the self (Barnes, 2018). Rewilding Britain (2023b) has a comprehensive glossary. Yet, however beneficial, rewilding is an anthropocentric response that maintains the status quo and is dependent on landowners and the government. As the Earth is critical to human survival, relational change necessitates an eco-centric, Earth-centered paradigm.

## Paradigm change

### Earth-centered arguments: relational aspects

The relational aspect of human–nature interactions informs my call for replacing an anthropocentric view with an eco-centric one. For Aline Lapierre, replacing therapeutic neutrality with a relational-centric therapy paved the way for embodied clinical understandings and active participation. She (LaPierre, 2015, p. 18) states that we exist in “a relational matrix within ourselves, with each other, and within our planet.” Arguing that humans are hardwired to connect, communicate, and make relationships, she uses body–mind theory to understand this complex relational matrix, outlining how humans reconcile inner and outer worlds through somatic (sensory), linguistic (narrative), and relational (attachment, identities, dyads) organizations. Disorganization in any of these forms is a rupture that limits development and results in withdrawal, an incapacity to heal therapeutically, to connect deeply, and to function relationally. This is precisely what eco-anomie looks like. Agency is compromised. The concerns of those who care deeply go unaddressed. Thus, individual and collective environmental actions that are suppressed by self-protective mainstream political and institutional culture become extreme, a polarization seen in protests by groups such as the Extinction Rebellion (2023) and democratic crises in the UK where political actions threaten human rights (Liberty, 2023).

### Earth-centered arguments: an ethics of balance

Planetary and health-based ecosystem concepts share similar goals around balance, achieving homeostasis, building functionality, and resilience. In addition to the need to theorize the concept that nature is in charge and not humans, it is important to consider the colonializing of peoples, taking care not to prioritize an anthropocentric mode. The Global North's mainstream political and economic culture informs and influences international policies, operating institutional power politics over the Global South and the planet. Its disproportionate contributions to climate change are, in no doubt. The Global South requires resources to implement appropriate science-based research that is not simply imposed from the Global North (Rodrigues, 2021). Anthropocentric processes require critique, and UK actions proceed more slowly than those of other countries (Monbiot, 2023). Protectionism in the North must not mean the South loses out again.

Designed as a collaborative and collective global community agreement with Earth in mind, the 17 United Nations Sustainable Development Goals (UNSDGs) called for change by 2030. Although collectively designed, they involve compromises between nations that operate within the dysfunctional power system and a “development” agenda (Sultana, 2018). From an earth-centered perspective they are not sustainable. They do not respect the fragile integrity of the Earth as a global system. Indeed, Kotzé and Adelman (2023) precisely argues that the SDGs are mainly human-focused rather than earth-centered and simply constitute the most recent anthropocentric agenda.

Tensions between positivist notions of absolute truth and human perceptions and experiences of the same have led to post-positivist framing and the development of systems-based approaches. However, like the SDGs, such improvements (and research funding) involve the agenda of an overarching anthropocentric paradigm driven by over-consumption and over-production. The earth's resources have been depleted by an economics of mismanagement, blocking future visions of more sustainable, healthy human–nature interaction. For some time, research and the United Nations have both acknowledged that burning fossil fuels is the biggest contributor to climate change (Karl and Trenberth, 2003). The second is agriculture and land use. Clearly, human management of earth resources cannot be trusted.

From the hedgerow, I observe the fundamental disconnect between humans and their knowledge/education (loss of capital + habitus) of the changing space that they/we inhabit (eco-anomie) and how to function within it (practice). This knowing-doing gap disrupts collective consciousness and actions, making it a problematic place from which to call for change. Yet for decades, individuals and communities of people have been calling. Governments need to act faster. Time is running out. A radical change of paradigm is urgently needed, where human interests no longer come first. The politics of human ownership over the earth do not serve our animal dependency on it. This requires that Earth law and rights come first (Cullinan, 2011).

## The new paradigm: Earth laws and wild law

Clearly, Earth laws are needed now to protect the planet and prevent further damage. Reframing Earthrights involves limiting human power and shifting perspective. Using combinations of natural law, ancient wisdom, and human and ecological laws, Cullinan (2011) advocates that the wellbeing of the planet is paramount. Human wellbeing cannot take precedence. He argues that Earth jurisprudence would bring animals back into the frame, meaning all animals would be equal. Land ownership and the purposes for which it is used would require revision. Time and natural cyclical rhythms need reprising to think differently about our short-term focus. Drawing inspiration from the practices of indigenous peoples, he compares human duty to the contributory role and allegiance of a cell to the health of a body, in an analogy that describes the Earth as a “vast network of interrelationships” (Cullinan, 2011, p. 101). Earth laws premise that all aspects of the Earth, air, and waters have rights relating to their nature, which is essentially wild. For example, it is the nature of rivers to flood. The flow of rivers would have rights requiring precedence over housing on flood plains. Wild laws such as these could inform Earth laws bringing balance back to the planet.

With the Earth laws foremost, information from Earth scientists and geographers who have identified climate change and ways of working with the Earth within the Anthropocene is needed. Observational science and other ways of knowing that Levi-Strauss called magical thinking are crucial, as are models from nature.

### Listening to the Earth

The Global North needs to learn from the Global South. In the Global North, Christianity has incorporated and transformed pagan knowledge, and science has eschewed indigenous practices in favor of epistemological knowledge (Glotfelty and Fromm, 1996). Many indigenous communities retain the ecological principles of working with and communicating with the Earth. These intuitive and magical practices are valuable for rethinking Earth laws, but they are fragmented or lost in the Global North. Bricolage can help create new narratives, and deep ecology and biophilia offer hope for relational functioning—that what has been lost may grow and resonate intuitively.

Scientists working with the Earth have learned useful forms of listening. Identifying human influences shaping the Earth and their potential to enable sustainable resources, Crutzen (2006) theorized the Anthropocene. Steffen's et al. (2005) contributions include initial empirical data analysis that informed current understanding of the Earth as a global integrated system and extrapolation of the Anthropocene. These cumulated in his understanding that environmental law had failed to limit or address damage and the need for Earth laws to challenge continuing political agendas (Steffen et al., 2005; Boonstra et al., 2023).

### Hope for creativity

Earth laws premise *returning to the bricolage*: Its creative process goes to the heart of ontological and existential questions about the purpose, meaning, and survival of human and planetary existence. Threads emerge in shreds and tatters as overarching politics maintains eco-anomie and climate change accelerates. Bricolage engages ontologically with this process to reconstitute a network of calls for action. Threads in vitalism and deep ecology sparkle with hope for change and food for future thought. Haraway's insects, fragments of tree worship, and early alphabets combine bits of myth and storying in this bricolage. Textures in philosophy, language, and research softly demonstrate that human–nature relations are integral to human flourishing. Integration requires altered perception and new thinking that involves experiencing, listening, and learning.

Individuals can enact care of the self:

- Spend more time in nature to come into being, to sustain health and nourish the soul;
- Actively seek creative interaction with the natural world;
- Become informed, recognize stasis, and develop Earth-centered actions;
- Eat well, exercise, develop community, and reduce over-consumption;
- Drive change by voting and making governments more accountable.

## Staying with the trouble: conclusion

Haraway has pointed out that all stories end badly in the Anthropocene. I have considered language, research, practices, and concepts in human–nature relationships. There are inevitable gaps, yet clear patterns of dis-ease have exposed the roots of a cultivated split identified as eco-anomie. The disconnected and unethical economy is the result of human mismanagement of the Earth, abusing resources to feed expansion, production, development, and ownership of land and peoples, with unhealthy results across all living systems and species. And as Haraway argues, we need to stay with the problem, perhaps drawing on old paradigm research, perhaps to generate and measure feedback systems as temporary Earth communications. There is no solution to climate change. All that is certain is that a new paradigm based on Earth laws is needed to regulate the political and economic mismanagement of our planet.

There is some hope that, as people have created the current problems, some mitigation may lie in healing people–nature relationships. Chawla's (2020) research on children caring for nature found their distress and sadness in coping with environmental loss and degradation may be helped by connecting with nature. Foucault's appraisal of Classical Stoicism also considers time in nature as a form of self-care and healing, especially for the human spiritual dimension.

Arguably, human–nature interactions form an intimate relationship like no other. Sensing or knowing when a plant is ready and how to gather sustainably, how to touch, taste, cut, and prepare, what and when to take and what to leave—all of this gives a rich and tangible practice of different and magical ways of knowing living assemblages that nurture. If the psyche is the breath of life and one that people share with the Earth, it may be emotions and senses that come to the rescue, but change needs to come quickly to enable people to embrace uncertainty.

## Author contributions

JP: Writing – original draft, Writing – review & editing.
